# Assessment of Counseling Self-Efficacy: Validation of the German Counselor Activity Self-Efficacy Scales-Revised

**DOI:** 10.3389/fpsyg.2021.780088

**Published:** 2021-12-16

**Authors:** Daniela Hahn, Florian Weck, Michael Witthöft, Franziska Kühne

**Affiliations:** ^1^Department of Clinical Psychology, Psychotherapy and Experimental Psychopathology, Psychological Institute, University of Mainz, Mainz, Germany; ^2^Department of Clinical Psychology and Psychotherapy, University of Potsdam, Potsdam, Germany

**Keywords:** counselor activity self-efficacy scales, counseling self-efficacy, psychotherapy training, assessment, factor structure, validation

## Abstract

**Background:** Many authors regard counseling self-efficacy (CSE) as important in therapist development and training. The purpose of this study was to examine the factor structure, reliability, and validity of the German version of the Counselor Activity Self-Efficacy Scales-Revised (CASES-R).

**Method:** The sample consisted of 670 German psychotherapy trainees, who completed an online survey. We examined the factor structure by applying exploratory and confirmatory factor analysis to the instrument as a whole.

**Results:** A bifactor-exploratory structural equation modeling model with one general and five specific factors provided the best fit to the data. Omega hierarchical coefficients indicated optimal reliability for the general factor, acceptable reliability for the *Action Skills-Revised* (AS-R) factor, and insufficient estimates for the remaining factors. The CASES-R scales yielded significant correlations with related measures, but also with therapeutic orientations.

**Conclusion:** We found support for the reliability and validity of the German CASES-R. However, the subdomains (except AS-R) should be interpreted with caution, and we do not recommend the CASES-R for comparisons between psychotherapeutic orientations.

## Introduction

Many authors regard counseling self-efficacy (CSE) as an important element of therapist development and training (e.g., Larson, 1998). CSE is an extension of Bandura’s (1986, 1997) social cognitive theory and can be defined as counselors’ beliefs about their “capabilities to effectively counsel a client in the near future” (Larson and Daniels, 1998, p. 180). This includes perceived abilities in therapy-related tasks, such as performing defined basic helping skills, managing the therapy session, or coping with challenging clinical situations (Lent et al., 2003). Larson (1998) assumed CSE to be relevant in, for example, counselors’ affective, cognitive, and behavioral reactions. For instance, counselors, with higher CSE beliefs in addition to at least adequate levels of counseling ability, might invest more effort in the face of difficult therapy situations and might be more composed within therapy sessions (Lent et al., 2006).

Regarding empirical results, in their review, Larson and Daniels (1998) reported associations of higher CSE ratings with greater positive and less negative (especially anxiety) affect with respect to the therapeutic role. Negative relations to physiological stress (Lannin et al., 2019) and positive relations with cognitive flexibility and emotional literacy (Asude and Zeynep, 2020) were also discussed. Additionally, several studies indicated positive relations of CSE with therapeutic and supervisory experience (e.g., Larson et al., 1992; Zhang et al., 2020). Nevertheless, there have been mixed results concerning counselor performance (e.g., Johnson et al., 1989; Larson et al., 1992). To further examine CSE and its relations to therapy-relevant variables, more research is necessary (Lent et al., 2006). For this purpose, a systematic and valid assessment of CSE is a prerequisite, and forms the focus of this article.

In attempting to refine the measurement of CSE, Lent et al. (2003) took into account various measurement-related concerns of existing instruments (e.g., conceptual problems; Lent et al., 1998), resulting in the development of the Counselor Activity Self-Efficacy Scales (CASES). The CASES (Lent et al., 2003) is a frequently used instrument and assesses perceived beliefs about one’s abilities to effectively perform various tasks or to deal with various situations in counseling during the next week. It consists of three scales: 1. *Helping Skill Self-Efficacy* (HS), 2. *Session Management Self-Efficacy* (SM), and 3. *Challenges Self-Efficacy* (CC). The design of the CASES incorporated a developmental perspective and was based on the authors’ conceptual synthesis of the helping skills model (Hill and O’Brien, 1999), further research (e.g., Larson and Daniels, 1998), and their own clinical experience. Regarding its factor structure, Lent et al. (2003) separately computed exploratory factor analyses (EFA) for the three parts and performed a second-order factor analysis. They extracted six first-order factors, two-second-order factors and, additionally, defined a total score. Concerning its psychometric properties, Cronbach’s *α* coefficients were acceptable to excellent (0.79 ≤ *α* ≥ 0.97) and the two-week test-retest reliability was adequate. Strong correlations with corresponding scales of the Counseling Self-Estimate Inventory (COSE, Larson et al., 1992) supported its convergent validity, while small to nonsignificant correlations with the Social Desirability Scale (Crowne and Marlowe, 1960, 1964) indicated its discriminant validity. The authors also reported on its criterion-related validity, i.e., negative correlations with negative affect and positive correlations with positive affect with respect to the counselor role (Positive and Negative Affect Schedule, PANAS; Watson et al., 1988). Furthermore, the CASES was sensitive to change over a one-semester internship and to different levels of counseling experience (Lent et al., 2003).

The CASES has been used in different cultural backgrounds (e.g., Lai et al., 2021) and a client-specific version of the instrument was investigated (Lent et al., 2006). But as far as the authors know, the factor structure was so far only reexamined in Turkish (Pamukçu and Demir, 2013). Pamukçu and Demir (2013) examined its dimensional structure and psychometric properties by analyzing the scales separately. The results provided further evidence to support the six first-order factors, and the authors defined a total score. McDonald’s Omega coefficients (*ω*) were acceptable to excellent (0.75 ≤ *ω* ≥ 0.98). Correlation patterns of the CASES with the COSE (Larson et al., 1992) provided further support for its convergent validity.

In research, the CASES has been applied in a variety of ways, using the individual scales (e.g., Chen et al., 2020; Ahn et al., 2021), the total score (e.g., Andersen et al., 2021; Meydan, 2021), or combinations (e.g., Lent et al., 2006; Ryba et al., 2021). However, if the goal is to measure general CSE by using the CASES total score, it is methodologically important to examine the dimensional structure of the instrument as a unit and to investigate whether specific facets explain sufficient variance beyond the general factor. More research is necessary concerning the factor structure, reliability, and validity of the CASES (e.g., Lent et al., 2003), so as to obtain further information on its appropriate application.

Furthermore, to gain a better understanding of the CASES in different cultures, more research is desirable. In Germany, there is currently no validated instrument to assess CSE. Additionally, as CSE is regarded as an important element of therapist development and training (e.g., Larson, 1998), its further examination in this context might enhance our understanding of CSE with respect to German psychotherapeutic trainees. Regarding Germany, the psychotherapy training and licensure are regulated by law (PsychTh-APrV, 1998; PsychThG, 2019). The admission requirements for psychotherapy training for adults are a master’s degree in psychology or medicine. Currently, training takes at least 3 years and includes more than 4,200 h, i.e., 600 h of theory, 1,200 of clinical work in a psychiatry clinic, 600 in a facility for psychosomatic care or psychotherapy, and 600 of outpatient treatment. Since the reform of the psychotherapy law in 2020, the requirement for psychotherapy training will be a master’s degree in the newly introduced psychotherapeutic study, which will take 5 years.

The first objective was the translation of the CASES into the revised German version (Counselor Activity Self-Efficacy Scales-Revised, CASES-R). The second objective was to examine the factor structure for the instrument as a whole and its internal consistencies. We expected the proposed factor structure reported by Lent et al. (2003) as a plausible solution. The third objective was to evaluate the validity of the CASES-R. Concerning the convergent validity, we expected the CASES-R scores to be significantly positively associated with general SE, and to an even greater extent with more domain-specific occupational SE (e.g., Bandura, 1997). In relation to the criterion validity, we hypothesized a positive relationship with positive affect and quality of the therapeutic relationship, and negative relations to negative affect. Regarding therapeutic characteristics, we assumed positive correlations between CASES-R ratings and therapeutic (years since psychotherapy training) and supervisory experience (completed supervision sessions). Furthermore, we explored the relationship between psychotherapeutic orientations (i.e., cognitive behavioral orientation and psychodynamic/psychoanalytic orientation) and the CASES-R.

## Materials and Methods

### Recruitment, Inclusion Criteria, and Sample

**Figure 1:**
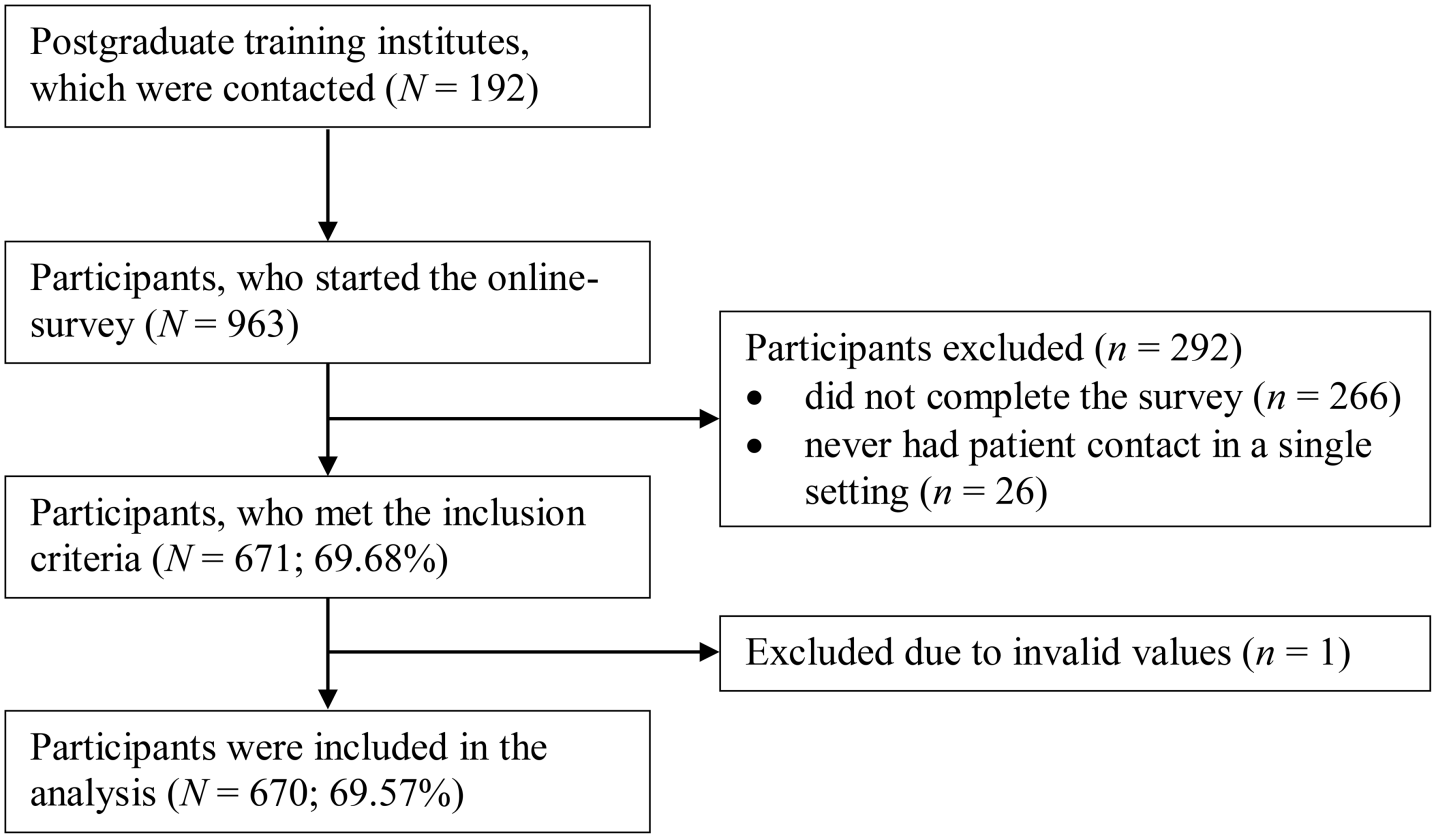
Flowchart of attrition.

The sample consisted of psychotherapy trainees who were recruited from postgraduate training institutes for adult psychotherapy throughout Germany. We contacted the training institutes by e-mail, asking them to distribute a link to their trainees, which led to the online survey. Participation was voluntary and anonymous. The inclusion criteria were (1) participation in psychotherapy training, (2) at least one patient contact in a single setting, (3) completion of the survey, and (4) giving informed consent. Figure 1 displays the participant flow. Characteristics of the final sample (*N* = 670) are displayed in Table 1. The Ethics Committee of the department of psychology at the Johannes Gutenberg-University Mainz approved the study (2017-JGU-psychEK-018).

**Table 1:** Characteristics of the total sample and separated according to trainees with cognitive behavioral orientation (CBT) and psychodynamic/psychoanalytic orientation (PT).

Characteristics	Total sample	CBT	PT
	(*N* = 670)	(*n* = 460)	(*n* = 204)
Age (*M*, *SD*)	31.70 (6.25)	31.15 (5.64)	33.03 (7.34)
Gender (*n*, %)			
female	575 (85.8%)	404 (87.8%)	166 (81.4%)
male	91 (13.6%)	55 (12.0%)	36 (17.6%)
other	4 (0.6%)	1 (0.2%)	2 (1.0%)
Psychotherapeutic orientation (*n*, %)			
Cognitive behavioral	460 (68.7%)	–	–
Psychodynamic therapy	153 (22.8%)	–	–
Psychoanalysis	46 (6.9%)	–	–
Psychodynamic therapy and psychoanalysis	5 (0.7%)	–	–
Other	6 (0.9%)	–	–
Years since psychotherapy training (*M*, *SD*)	3.10 (2.35)^1^	3.09 (2.28)	3.19 (2.50)^1^
Completed supervision sessions (*M*, *SD*)	57.21 (62.73)	55.70 (54.25)	61.81 (78.94)

1 *Due to unclear information n = 1 missing*.

### Measures

In order to collect data, such as age and variables of psychotherapeutic experience, the survey included a brief sociodemographic questionnaire.

#### Counselor Activity Self-Efficacy Scales

The CASES (Lent et al., 2003) assesses CSE in three scales: HS, SM, and CC. It consists of 41-items and uses a 10-point self-report scale, ranging from 0 (*no confidence at all*) to 9 (*complete confidence*). Higher scores reflect higher CSE.

The HS scale measures perceived capabilities in performing relatively structured, primary skill elements and consists of three factors: (1) *Exploration Skills* (5 items), i.e., basic communication skills (e.g., “restatements”), (2) *Insight Skills* (6 items) depict skills to help clients gain an understanding of their problems (e.g., “interpretations”), and (3) *Action Skills* (4 items), which represent skills in applying relatively structured interventions (e.g., “direct guidance”).

The SM scale is unidimensional and assesses the perceived ability to manage therapy sessions (e.g., Keep sessions “on track” and focused.).

Third, the CC scale measures the perceived ability to handle more advanced or challenging clinical situations regarding two factors: (1) *Relationship Conflict* (10 items), which describes skills in managing difficult interpersonal situations within therapy sessions (e.g., a client who “is at an impasse in therapy”) and (2) *Client Distress* (6 items) representing skills in working with challenging therapy situations (e.g., a client who “is suicidal”).

For the purposes of this study, we translated the CASES into German, following an elaborate adaption process (Wild et al., 2005). Steps included two forward translations, reconciliation, one back translation of a native-speaking clinical psychologist, a review and harmonization of the results, cognitive debriefing (*n* = 10), proofreading by an independent psychologist, and finalization. The term “counselor” was changed into “therapist.”

#### Convergent Validity

We used the German version of the *General Self-Efficacy Scale* (GSE; Schwarzer and Jerusalem, 1999) to assess general SE, i.e., an optimistic sense of personal competence. The unidimensional, 10-item scale includes positive statements about general personal capabilities (e.g., “Thanks to my resourcefulness, I know how to handle unforeseen situations.”). The GSE uses a 4-point Likert scale, ranging from 1 (*not at all true*) to 4 (*exactly true*). Higher values reflect higher general self-efficacy. In the present study, Cronbach’s *α* was 0.83.

To assess occupational self-efficacy, we used the German short version of the *Occupational Self-Efficacy Scale* (OCCS; Rigotti et al., 2008). The unidimensional, 6-item scale refers to the belief in ones’ own ability to successfully fulfill work-related tasks (e.g., “I can remain calm when facing difficulties in my job because I can rely on my abilities.”). The OCCS uses a 6-point Likert scale, ranging from 1 (*not at all true*) to 6 (*completely true*). Higher scores reflect higher occupational self-efficacy. Cronbach’s *α* was 0.86 in the current sample.

#### Criterion Validity

We used the total score of the Irritation Scale (e.g., Müller et al., 2004) to assess emotional (e.g., “When I come home tired after work, I feel rather irritable.”) and cognitive (e.g., “Even at home I often think of my problems at work”) strain in working contexts. The Irritation Scale consists of 8-items and uses a 7-point self-report scale, ranging from 1 (*strongly disagree*) to 7 (*strongly agree*). Higher values reflect a higher level of irritation. In the present study, Cronbach’s *α* was 0.87 for the total score.

To assess the trainee’s affective state, the PANAS (Watson et al., 1988; German version: e.g., Krohne et al., 1996 Breyer and Bluemke, 2016) was used. The 20-item scale is divided into the dimensions of positive affect (PA; e.g., “excited”) and negative affect (NA; e.g., “distressed”). The PANAS uses a 5-point Likert scale, ranging from 1 (*not at all true*) to 5 (*extremely*). Higher values indicate a higher extent of the corresponding affective dimension. Based on Lent et al. (2003), we adapted the instruction to link the affect ratings to the therapeutic role (e.g., “in general, as a therapist, …”). In the current study, Cronbach’s *α* coefficients of 0.81 (PA) and 0.78 (NA) were obtained.

We further used the Helping Alliance Questionnaire (HAQ; Luborsky, 1984; German version: Bassler et al., 1995) to assess the quality of the therapeutic alliance. The HAQ consists of 11 items and uses a 6-point Likert scale, ranging from 1 (*strongly disagree*) to 6 (*strongly agree*). The HAQ was provided in a therapist version (HAQ-T) and to fit the study context, it was adapted to patients in general. The instruction reads “Please rate the therapeutic alliance with respect to your patients in general ….” The Cronbach’s *α* of the total scale was 0.85.

### Data Analysis

For data analysis, we used IBM SPSS Statistics (Version 23), M*plus* (Version 5; Muthén and Muthén, 1998–2010), and R (Version 4.0.0; R Core Team, 2020). We applied M*plus* to examine the factor structure, and R to compute omega hierarchical coefficients using the Bifactor Indices Calculator (Dueber, 2020). The significance level was set to *α* = 0.05. Apart from one unclear information in the sociodemographic data section (see Table 1), the data set did not contain any missing data.

### Model Fit Evaluation

We evaluated the model fit using the *χ*2-test, the ratio of *χ*2/*df*, the comparative fit index (CFI), the Tucker-Lewis Index (TLI), the standardized root mean square residual (SRMR), and the root mean square error of approximation (RMSEA). We applied generally accepted standards regarding the model fit (e.g., Hu and Bentler, 1999; Yu, 2002; Schermelleh-Engel et al., 2003). Nonsignificant results for the *χ*2-test indicate an adequate fit. For the ratio of *χ*2/*df*, values below 3 suggest an acceptable fit, and values below 2 indicate a good fit (Bollen and Long, 1993). CFI and TLI values greater than 0.90 indicate an acceptable fit, and values greater than 0.95 indicate a good fit. SRMR values should remain less than 0.08. For the RMSEA, values lower than 0.08 indicate an acceptable, and below 0.05, a good model fit. For the comparison of models, we computed the *χ*2-difference test using the Satorra-Bentler Scaled *χ*2.

### Factor Structure

We first computed confirmatory factor analyses (CFAs) by applying the robust maximum likelihood estimator with the total sample, in order to investigate the original factor structure for the whole instrument. For this purpose, we examined a correlated factor model with six correlated latent variables (Model 1), a hierarchical factor model with six correlated first-order factors and one-second-order factor (Model 2a), and two-second-order factors (Model 2b). In the case of no acceptable model fits, we randomly divided the total sample into two subsamples (*n*_1_ and *n*_2_), applying the relevant SPSS function. With the first subsample *n*_1_, we performed an EFA using principal axis factoring (PAF) with oblique rotation (promax) to investigate the measurement structure. With the second subsample *n*_2_, we further examined the resulting factor structure of the EFA using different CFA models, including bifactor (e.g., Reise, 2012) and exploratory structural equation modeling (ESEM; e.g., Asparouhov and Muthén, 2009). For the investigation of the measurement models, we followed the recommendations of Morin et al. (2016).

### Reliability

We evaluated internal consistencies computing omega hierarchical coefficients using the Bifactor Indices Calculator in R (Dueber, 2020). Omega coefficients from 0.50 are acceptable, and from 0.75 optimal (Reise et al., 2013).

### Construct Validity and Trainee Experience

We calculated Pearson’s correlations between sufficient reliable CASES-R scales, related constructs (GSE, OCCS, IR, modified PANAS, and modified HAQ-T), and trainee experience (therapeutic and supervisory experience). Correlations were compared using Fisher’s (1925) *z* transformation.

### Relationship Between Therapeutic Orientation and the CASES-R

For the comparisons of groups, and due to the small sample size, we excluded other forms of therapy (*n* = 6), and pooled participants from psychoanalytical and/or psychodynamic orientations into one group (PT; *n*_PT_ = 204). We then contrasted PT with cognitive-behavioral participants (CBT; *n*_CBT_ = 460). Table 1 displays characteristics of the total sample and of the CBT and PT subsamples. We analyzed differences in terms of dimensional variables (e.g., age) using *t*-tests and examined gender using *Φ*-test (excluding other *n* = 4). Point-biserial correlation coefficients were computed between therapeutic orientation (0 = CBT, 1 = PT) and the CASES-R. In the case of sociodemographic differences, we conducted a hierarchical multiple regression analysis. We defined CSE as the dependent variable and entered the predictor variables in two steps as follows. In step 1, we entered the corresponding sociodemographic variables, and in step 2, we entered the therapeutic orientation.

## Results

### Characteristics of the Participants

Characteristics of the total sample and separated according to CBT and PT trainees are shown in Table 1. PT trainees were on average 1.88 years older, *t*(313.58) = −3.27; *p* = 0.001; *d* = −0.29, and more often male, *Φ* = −0.08; *p* = 0.05, than CBT trainees. They did not differ regarding therapeutic, *t*(661) = −0.48; *p* = 0.63, and supervisory experience, *t*(291.31) = −1.01; *p* = 0.32.

### Confirmatory Factor Analysis

The CFAs for the correlated six-factor solution (Model 1) did not adequately fit the data for most indicators (Table 2). Even when taking the hierarchical nature of the instrument into account by considering one (Model 2a) and two (Model 2b) second-order factors, the data still demonstrated suboptimal fits in terms of most indicators.

**Table 2:** Goodness-of-fit statistics for competing factor structure models of the Counselor Activity Self-Efficacy Scales-Revised (CASES-R).

Model	*χ* ^2^	*df*	*χ* ^2/*df*^	CFI	TLI	RMSEA	SRMR	*χ* ^2^_diff_	*df*_diff_
1. Correlated Factor Model^1^ total sample; 6 factors	2860.763***	764	3.744	0.800	0.786	0.064	0.065	–	–
2a. Hierarchical CFA^1^ total sample; 1 s-order factor, 6 factors	2953.706***	773	3.821	0.792	0.780	0.065	0.069	–	–
2b. Hierarchical CFA^1^ total sample; 2 s-order factors, 6 factors	2907.617***	772	3.766	0.792	0.780	0.065	0.069	–	–
3. Single CFA *n*_2_; 1 factor	1358.317***	324	4.192	0.666	0.638	0.098	0.088	907.077***	120
4. Correlated CFA *n*_2_; 5 factors (EFA)	618.357***	314	1.969	0.902	0.890	0.054	0.055	254.996***	110
5. Hierarchical CFA n_2_; 5 factors (EFA), 1 s-order factor	639.629***	319	2.005	0.896	0.886	0.055	0.060	275.047***	115
6. Bifactor CFA *n*_2_; 5 factors (EFA), 1 global factor	550.842***	297	1.855	0.918	0.903	0.051	0.053	192.594***	93
7. ESEM *n*_2_; 5 factors (EFA solution); target rotation; cross-loadings allowed	470.934***	226	2.084	0.921	0.877	0.057	0.034	127.217***	22
8. Bifactor-ESEM *n*_2_; 5 factors (EFA), 1 global factor; target rotation; cross-loadings allowed	355.330***	204	1.742	0.951	0.916	0.047	0.028		

1 According to Lent et al., 2003; CFA = confirmatory factor analysis; ESEM = exploratory structural equation modeling; *df* = degrees of freedom; CFI = comparative fit index; TLI = Tucker-Lewis index; RMSEA = root mean square error of approximation; *χ*2_diff_ = chi-square difference test for the comparison of the models; and *df*_diff_ = degrees of freedom of the chi-square difference test. Application of the robust maximum likelihood estimator (MLR). ESEM was estimated using target oblique rotation; Bifactor-ESEM was estimated using bifactor orthogonal target rotation.

*** *p* < 0.001.

### Exploratory Factor Analysis

Since the CFAs did not lead to a satisfactory solution, the total sample was randomly divided into two subsamples (*n*_1_ = 336, *n*_2_ = 334). No significant differences between the two subsamples *n*_1_ and *n*_2_ were observed for age, *t*(668) = −0.28, *p* = 0.78, gender, *χ*2(1) = 0.28, *p* = 0.59, psychotherapy orientation, *χ*2(1) = 0.01, *p* = 0.92, therapeutic, *t*(667) = −0.39, *p* = 0.70, and supervisory experience, *t*(668) = −0.70, *p* = 0.49.

We performed an EFA using PAF with oblique rotation (promax) on the 41 items with *n*_1_. The Kaiser-Meyer-Olkin measure (KMO = 0.90; Hutcheson and Sofroniou, 1999) and Bartlett’s test of sphericity, *χ*^2^(820) = 6951.92; *p* < 0.001, signified the adequacy of the analysis. Eight factors showed eigenvalues greater than 1 (Kaiser’s criterion). A review of the scree plot (Cattell, 1966) was ambiguous and justified one, three, five, or eight factors. Parallel analysis yielded five factors (Horn, 1965; O’Connor, 2000). Considering the interpretability of the solutions and the parallel analysis, we retained five factors for the final model. Items (Part. Item) were eliminated in two steps. Two items (1.7 and 1.11) failed to load sufficiently on a factor (<0.40), 8 items (1.4, 1.6, 1.10, 1.12, 2.7, 2.10, 3.6, and 3.10) showed substantial cross-loadings (>0.30) on another factor, and four items (2.3, 2.4, 3.1, and 3.5) were rejected due to the interpretability of the solution. Supplementary Table A displays factor loadings after rotation. We labeled the revised instrument CASES-R and termed the specific factors as “*Relationship Conflict-Revised*” (RC-R; 9 items), “*Session Management-Revised*” (SM-R; 6 items), “*Exploration and Insight Skills-Revised*” (EIS-R; 6 items), “*Client Distress-Revised*” (CD-R; 3 items), and “*Action Skills-Revised*” (AS-R; 3 items).

### Confirmatory and Exploratory Structural Equation Measurement Approaches

To further examine the measurement structure of the CASES-R, we applied confirmatory and exploratory structural equation measurement approaches with sample *n*_2_ (Table 2). A single-factor solution (Model 3) demonstrated a poor fit across the indicators. A hierarchical solution (Model 5) showed a better but still suboptimal fit. A correlated solution (Model 4) and an ESEM model (Model 7) showed an improved fit, which was further improved by the bifactor model (Model 6). Also, the *χ*2 difference test was significant for all tested comparisons. The bifactor-ESEM model fitted the data significantly better than the other models (Table 2). Supplementary Table B presents the standardized parameter estimates of the bifactor-ESEM model.

### Reliability

Omega hierarchical coefficients (Table 3) indicated, for the total score, an optimal, for the AS-R an acceptable, but for the other four specific factors an unacceptably low (all <0.50) reliability.

**Table 3:** Means, standard deviations, and omega hierarchical coefficients for the CASES-R in the total sample (*N* = 670).

Factors	*M*	*SD*	ωH
Exploration and Insight Skills-Revised	7.19	1.03	0.36
Action Skills-Revised	5.58	2.19	0.64
Session Management-Revised	6.05	1.36	0.31
Client Distress-Revised	5.35	1.90	0.43
Relationship Conflict-Revised	5.12	1.36	0.32
CASES-Revised Total Score	5.87	1.07	0.79

ωH = Omega hierarchical; higher scores indicate higher counseling self-efficacy (0 = no confidence at all and 9 = complete confidence).

### Construct Validity and Trainee Experience

The correlations between CASES-R scales and related constructs ranged from small to high (Table 4). The correlation between GSE and OCCS (*r* = 0.67; *p* < 0.001) differed significantly for the CASES-R total score (*z* = −3.33, *p* < 0.001), while we observed no significant difference for AS-R (*z* = 1.30, *p* = 0.19). The correlations between CASES-R scales and trainee experiences (Table 4) were not significant to small.

**Table 4:** Correlations of the CASES-R total score and Action Skills-Revised (AS-R) subscale to related measures and therapists’ characteristics.

		Total sample (*N* = 670)	
Measures		CASES-R total Score	AS-R
Convergent validity	GSE	0.43^b^,***	0.21^b^,***
	OCCS	0.52^b^,***	0.17^b^,***
Criterion validity	PANAS-PA	0.41^b^,***	0.19^b^,***
	PANAS-NA	−0.26^b^,***	−0.16 ^b^***
	IR	−0.28^b^,***	−0.13^b^,***
	HAQ-T	0.61^b^,***	0.25^b^,****
Years psychotherapy training^1^		0.15^b^ ^,^***	0.03^b^
Completed supervision sessions		0.17^b^ ^,^***	−0.01^b^
Therapeutic orientation (0 = CBT, 1 = PT)		−0.12^a^ ^,^**	−0.50^a^ ^,^***

** *p* ≤ .01; ^*^p ≤ 0.05;

*** *p* ≤ 0.001.

1 Due to unclear information *n* = 1 missing.

a Point-biserial correlation coefficient.

b Pearson correlation coefficient.

GSE = General Self-Efficacy; OCCS=Occupational Self-Efficacy; IR = Irritation Scale; PANAS = Positive and Negative Affect Schedule; PA = Positive Affect; NA = Negative Affect; and HAQ-T = Helping Alliance Questionnaire-therapist version. CBT = Cognitive-behavioral orientation; PT = Psychodynamic/Psychoanalytic orientation.

### Relationship Between Therapeutic Orientation and the CASES-R

Correlations between the CASES-R and psychotherapeutic orientation were small for the total score and high for AS-R (Table 4). The multiple regression analyses showed, in step 2, by entering the variable of psychotherapeutic orientation, a significant change in *R^2^* for the total score, Δ*R*^2^ = 0.02; Δ*F*_(1, 657)_ = 14.74, *p* < 0.001, and AS-R, Δ*R*^2^ = 0.25; Δ*F*_(1, 657)_ = 218.11, *p* < 0.001. The variable psychotherapeutic orientation contributed significantly to the explanation of CSE (total score: *β* = −0.15, *p* < 0.001; AS-R: *β* = −0.50, *p* < 0.001).

## Discussion

Counseling self-efficacy represents a crucial construct in therapist training and development. A reliable and valid assessment of CSE are a prerequisite for future research in this area. The present article focuses therefore on the translation of the CASES into German and the examination of its factor structure, reliability, and validity.

Contrary to our hypotheses, the original 6-factor structure of the CASES (Lent et al., 2003) could not be replicated. Lent et al. (2003) examined the CASES scales separately, due to the three-part scheme underlying its construction and the proposed possibility of a separate use of the scales. However, as the CASES can also be used as a whole, and a total score can be calculated (Lent et al., 2003), it is important to also examine the CASES as a unit to gain a better understanding of its underlying dimensional structure. Analyzing the instrument as a whole, as well as the application of more conservative statistical requirements (especially parallel analysis), provided support for a five-factor solution.

In summary, a bifactor-ESEM model with one general and five specific factors yielded the best fit to our data. While the EIS-R factor represents a combination of the original *Exploration Skills* and *Insight Skills* factor, the other four specific factors (i.e., AS-R, SM-R, RC-R, and CD-R) now represent shorter versions of the original ones. Furthermore, our results support a general factor of CSE. However, even in the bifactor-ESEM model, some items of the EIS-R, SM-R, CD-R, and RC-R factors showed no substantial target loadings (e.g., 1.8 and 3.3) or significantly negative cross-loadings (e.g., 3.14), which may explain the unsatisfactory specificity of these subdomains. Nonetheless, these items had strong loadings on the general factor, suggesting that they are valuable indicators of general CSE.

Reliability estimates of the new factor structure yielded an optimal omega hierarchical coefficient for the general CASES-R factor and an acceptable one for AS-R. However, the other specific factors showed unacceptable low omega hierarchical coefficients, indicating that these factors may not explain sufficient variance beyond the total score. The feasibility of a separate interpretation of these factors is therefore in doubt, and only the general and the AS-R factor should be interpreted individually.

We found evidence to support the convergent and criterion validity of the CASES-R. In terms of the CASES-R total score, as hypothesized, we observed moderate correlations with general self-efficacy and significantly higher correlations with occupational self-efficacy. As anticipated, we observed positive correlations with positive affect, and negative correlations with negative affect and irritation. These results were in line with previous studies (e.g., Lent et al., 2003; Shoji et al., 2016). Additionally, as expected, the results showed positive relations with therapeutic alliance. In accordance, positive relations of CSE to counselor’s session quality ratings were also reported (e.g., Lent et al., 2006) and therapists with low or medium client-specific CSE underrated the working alliance regarding goals/tasks and bond more, than therapists with high client-specific CSE (Lai et al., 2021). The results also showed positive correlations with therapeutic and supervisory experience, which are consistent with previous studies (e.g., Larson et al., 1992). However, the correlations were only small. The reasons might be that these experiences do not necessarily contain sources of CSE (e.g., mastery experience; Larson, 1998) and might be influenced by additional factors, such as the supervisory working alliance (e.g., Morrison and Lent, 2018). Also, these results might be due to the relations to psychotherapeutic orientation (see below). Regarding the AS-R factor, as expected, we found similar relations with the examined constructs, but to a lesser extent. In addition, the correlations between occupational and general self-efficacy did not differ, and factors of trainee experience showed no correlations with AS-R. Besides its greater domain-specificity, this might also be due to its relations to psychotherapeutic orientation.

We found small correlations between the CASES-R total score and therapeutic orientation, as well as strong correlations between AS-R and therapeutic orientation. The results imply that CBT trainees tended to have higher values on these scales than the PT trainees. Interestingly, the items of the AS-R factor were based on the *Action Stage* of the *Helping Skills* model (Hill and O’Brien, 1999). Behavioral theories (e.g., Goldfried and Davison, 1994; for more details see Hill, 2014) formed the foundation of the action stage. Consequently, these items might be less responsive to PT trainees. At the same time, only two items from the former *Insight Skills* scale, which was derived from psychoanalytic and interpersonal theories (Lent et al., 2003; Hill, 2014), were retained within the CASES-R total score. This may have further promoted differences regarding psychotherapeutic orientations. It remains unclear whether the AS-R factor is adequate for measuring CSE in therapeutic orientations which do not emphasize behavioral theories. Thus, in future research, it would be useful to examine the suitability of the CASES-R in other psychotherapeutic orientations, such as family therapy. Meanwhile, we do not recommend using the CASES-R for comparisons between therapeutic orientations.

### Limitations

The present study aims to examine the factor structure and psychometric properties of the CASES-R. Nonetheless, there are several factors limiting interpretation.

As participants were German psychotherapeutic trainees and we did not collect information on the participants’ cultural background, there is a limit to the generalizability of the sample. Future studies should investigate the CASES-R in different cultural contexts and with varying participants therapeutic experience (e.g., licensed psychotherapists) and theoretical orientations.

Further investigation of the discriminant validity is also desirable. The observed higher correlation with the more specific occupational self-efficacy instrument, in comparison with general self-efficacy for the CASES-R total score, may be an indicator of the discriminant value of the CASES-R (e.g., Schwarzer and Jerusalem, 1999). Future research should also examine whether the inclusion of further items (e.g., additional items based on counseling behavior of effective therapists, such as interpersonal skills; e.g., Heinonen and Nissen-Lie, 2020), broaden our understanding of counselors’ perspective and its influence on therapy-relevant indicators (Lent et al., 2003).

Additionally, the survey was cross-sectional and only provides a broad estimate of the current CSE. Further research with longitudinal designs appears important in order to better understand the development of CSE within the context of ongoing psychotherapy training or to investigate variables (e.g., supervisory alliance) that might influence individual CSE (Lent et al., 2003).

Finally, the trainee perspective served as the basis for assessing CSE, but CSE is not equivalent to actual skills (Bandura, 1997). Moreover, evidence was found of therapist’s difficulties in adequately evaluating their own skills (Walfish et al., 2012). Greatly over- or underestimating of CSE might lead to negative implications for themselves or others, when, i.e., taking on activities for which they are ill prepared (Bandura, 1986). For example, overestimation of CSE for a specific patient might result in a neglection of problems (Lai et al., 2021). In the context of self-confidence, Nissen-Lie et al. (2017) reported that “professional self-doubt” was related to better client outcomes in the case of experienced therapists with a high degree of self-affiliation, which the authors describe as a lasting tolerant and nurturing central element in the personal-self. In contrast, Odyniec et al. (2019) described better client outcomes for more confident CBT trainees, as opposed to those who experienced more “professional self-doubt.” According to Bennett-Levy (2019), self-confidence and self-affiliation may be important for both inexperienced and experienced therapists, but a certain amount of “healthy self-doubt” might be needed. In summary, including different perspectives (e.g., Lent et al., 2003), such as therapists with different levels of skill, independent raters of therapy competence (e.g., Lent et al., 2003; Weck et al., 2017), or supervisors and clients (e.g., Lent et al., 2006), seems valuable for future studies examining CSE. Additionally, further research on the relations between global and client-centered CSE using the CASES-R (Lent et al., 2006) is desirable.

### Conclusion

In conclusion, the present study has important implications for both psychotherapy research and training. We examined the latent structure and psychometric properties of the CASES-R as a whole. The large sample of trainees and the statistical analyses are strengths of this study. However, the psychometric properties of the CASES-R warrant future research, for instance, with different psychotherapeutic orientations.

For psychotherapy training, the CASES-R could provide a useful instrument for educators and trainees to mutually reflect on CSE. A better understanding of trainees’ beliefs about their ability to perform therapeutic behaviors and to deal with various challenging therapeutic situations may in turn facilitate tailored and specific feedback, for example, in the context of supervision, and support self-reflection on CSE for fostering skills development.

## Data Availability Statement

The raw data supporting the conclusions of this article will be made available by the authors, without undue reservation.

## Ethics Statement

The studies involving human participants were reviewed and approved by the Ethics Committee of the Department of Psychology at the Johannes Gutenberg-University Mainz (application 2017-JGU-psychEK-018). The patients/participants provided their written informed consent to participate in this study.

## Author Contributions

FW, FK, and DH contributed to conceptualization and methodology. DH analyzed the data and wrote the first draft of the manuscript. MW contributed to methodology and analysis. FW and MW provided the resources. FK and FW supervised the project. FW administrated the project. All authors contributed to manuscript revision, and read and approved the submitted version.

## Conflict of Interest

The authors declare that the research was conducted in the absence of any commercial or financial relationships that could be construed as a potential conflict of interest.

## Publisher’s Note

All claims expressed in this article are solely those of the authors and do not necessarily represent those of their affiliated organizations, or those of the publisher, the editors and the reviewers. Any product that may be evaluated in this article, or claim that may be made by its manufacturer, is not guaranteed or endorsed by the publisher.
