# Supplementary material for: Assessment of Counseling Self-Efficacy: Validation of the German Counselor Activity Self-Efficacy Scales-Revised
Source: Front Psychol. 2021 Dec 16;12:780088. doi: 10.3389/fpsyg.2021.780088 (PMC8716828; doi:10.3389/fpsyg.2021.780088)
Supplement: Supplementary file 1 [file Table_1.docx]

***Supplementary Material 1***

**Supplementary Table A**

*Rotated Factor Loadings of the Counselor Activity Self-Efficacy Scales-Revised (n_1_= 336)*

| Part.Item | RC-R | SM-R | EIS-R | | CD-R | | AS-R |
| --- | --- | --- | --- | --- | --- | --- | --- |
| 1.1. | -0.053 | -0.121 | **0.604** | | 0.019 | | 0.148 |
| 1.2. | -0.054 | -0.030 | **0.668** | | -0.001 | | 0.047 |
| 1.3. | 0.083 | 0.035 | **0.537** | | -0.031 | | 0.015 |
| 1.5. | 0.032 | 0.119 | **0.612** | | 0.101 | | -0.140 |
| 1.8. | 0.087 | 0.238 | **0.461** | | -0.058 | | 0.033 |
| 1.9. | 0.056 | 0.120 | **0.593** | | -0.035 | | -0.022 |
| 1.13. | 0.031 | -0.036 | 0.138 | | -0.052 | | **0.621** |
| 1.14. | -0.044 | 0.056 | 0.025 | | 0.154 | | **0.657** |
| 1.15. | 0.036 | 0.009 | -0.035 | | -0.082 | | **0.911** |
| 2.1. | -0.072 | **0.639** | 0.116 | | -0.073 | | -0.124 |
| 2.2. | -0.049 | **0.623** | 0.106 | | 0.099 | | -0.014 |
| 2.5. | 0.028 | **0.658** | 0.062 | | 0.042 | | 0.046 |
| 2.6. | -0.004 | **0.707** | -0.050 | | 0.056 | | 0.104 |
| 2.8. | -0.091 | **0.740** | 0.003 | | 0.002 | | 0.111 |
| 2.9. | 0.032 | **0.889** | -0.093 | | -0.063 | | -0.048 |
| 3.2. | -0.082 | -0.018 | 0.070 | | **0.839** | | -0.031 |
| 3.3. | 0.161 | 0.099 | -0.090 | | **0.535** | | 0.006 |
| 3.4. | 0.007 | -0.037 | -0.015 | | **0.947** | | 0.017 |
| 3.7. | **0.619** | -0.140 | 0.015 | | -0.041 | | 0.069 |
| 3.8. | **0.549** | 0.097 | 0.034 | | 0.079 | | -0.004 |
| 3.9. | **0.488** | 0.030 | 0.062 | | -0.092 | | 0.006 |
| 3.11. | **0.584** | 0.002 | -0.155 | | 0.026 | | 0.172 |
| 3.12. | **0.644** | -0.186 | 0.051 | | 0.063 | | -0.094 |
| 3.13. | **0.759** | -0.001 | 0.043 | | -0.054 | | -0.076 |
| 3.14. | **0.665** | 0.234 | -0.165 | | -0.007 | | -0.101 |
| 3.15. | **0.468** | 0.011 | 0.155 | | -0.033 | | 0.091 |
| 3.16. | **0.556** | -0.058 | 0.064 | | 0.102 | | 0.036 |
|  | |  |  |  | |  |  |

*Notes*. Exploratory factor analysis was performed with principal-axis factoring procedures (PAF) and oblique rotation (promax). RC-R = Relationship Conflict-Revised; SM-R = Session Management-Revised; EIS-R = Exploration and Insight Skills-Revised; CD-R = Client Distress-Revised; AS-R = Action Skills-Revised. Counselor Activity Self-Efficacy Scales from Lent, Hill & Hoffman, 2003.
