# Supplementary material for: Assessment of Counseling Self-Efficacy: Validation of the German Counselor Activity Self-Efficacy Scales-Revised
Source: Front Psychol. 2021 Dec 16;12:780088. doi: 10.3389/fpsyg.2021.780088 (PMC8716828; doi:10.3389/fpsyg.2021.780088)
Supplement: Supplementary file 2 [file Table_2.docx]

***Supplementary Material 2***

**Supplementary Table B**

*Standardized factor loadings from the Bifactor-ESEM model (n_2_=334)*

| Part.Items | CASES-R | EIS-R | AS-R | SM-R | CD-R | RC-R |
| --- | --- | --- | --- | --- | --- | --- |
| 1.1 | **0.376***** | **0.381***** | 0.157 | -0.086 | -0.043 | 0.107 |
| 1.2 | **0.425***** | **0.428***** | 0.061 | -0.008 | -0.029 | -0.081 |
| 1.3 | **0.456***** | **0.481***** | 0.102 | 0.092 | 0.035 | -0.032 |
| 1.5 | **0.516***** | **0.619***** | -0.073 | 0.120* | 0.016 | -0.027 |
| 1.8 | **0.584***** | **0.289***** | 0.013 | 0.143 | 0.023 | -0.033 |
| 1.9 | **0.568***** | **0.397***** | -0.036 | 0.092 | 0.019 | -0.02 |
| 1.13 | **0.358***** | 0.068 | **0.600***** | 0.076 | -0.001 | -0.016 |
| 1.14 | **0.392***** | 0.079 | **0.618***** | 0.097 | 0.053 | -0.052 |
| 1.15 | **0.370***** | -0.013 | **0.760***** | 0.03 | -0.032 | -0.058 |
| 2.1 | **0.549***** | 0.014 | 0.019 | **0.392***** | -0.056 | -0.063 |
| 2.2 | **0.585***** | 0.104 | -0.069 | **0.551***** | 0.039 | -0.04 |
| 2.5 | **0.601***** | 0.093 | 0.007 | **0.450***** | -0.022 | -0.053 |
| 2.6 | **0.554***** | 0.087 | 0.199*** | **0.422***** | -0.068 | 0.058 |
| 2.8 | **0.564***** | 0.014 | 0.136** | **0.415***** | 0.09 | -0.01 |
| 2.9 | **0.554***** | 0.026 | 0.034 | **0.475***** | 0.061 | 0.031 |
| 3.2 | **0.600***** | 0.058 | -0.087*** | -0.034 | **0.511***** | 0.035 |
| 3.3 | **0.687***** | 0.026 | 0.031 | 0.011 | **0.220***** | 0.119* |
| 3.4 | **0.628***** | -0.035 | 0.068 | 0.063 | **0.672***** | 0.071* |
| 3.7 | **0.250**** | 0.044 | 0.043 | -0.001 | 0.055 | **0.693***** |
| 3.8 | **0.440***** | 0.055 | 0.05 | 0.012 | 0.066 | **0.360***** |
| 3.9 | **0.415***** | -0.084 | 0.068 | 0.04 | -0.03 | **0.343***** |
| 3.11 | **0.504***** | -0.055 | 0.003 | -0.149 | 0.013 | **0.077***** |
| 3.12 | **0.308***** | -0.018 | -0.087 | 0.077 | 0.112* | **0.689***** |
| 3.13 | **0.658***** | -0.004 | -0.199*** | -0.190** | -0.072 | **0.227***** |
| 3.14 | **0.744***** | -0.209*** | -0.140** | -0.097 | -0.078 | **0.054***** |
| 3.15 | **0.563***** | -0.019 | -0.123** | -0.107 | -0.069 | **0.354***** |
| 3.16 | **0.667***** | -0.114 | -0.005 | -0.031 | 0.028 | **0.262***** |

*Notes*. CASES-R = Counselor Activity Self-Efficacy Scales-Revised; ESEM = Exploratory structural equation model; EIS-R = Exploration and Insight Skills-Revised; AS-R = Action Skills-Revised; SM-R = Session Management-Revised; CD-R = Client Distress-Revised; RC-R = Relationship Conflict-Revised. Target factor loadings are highlighted in bold. *p≤.05; ** p≤.01; ***p≤.001.
